# Unconjugated Bile Acids Influence Expression of Circadian Genes: A Potential Mechanism for Microbe-Host Crosstalk

**DOI:** 10.1371/journal.pone.0167319

**Published:** 2016-12-01

**Authors:** Kalaimathi Govindarajan, John MacSharry, Patrick G. Casey, Fergus Shanahan, Susan A. Joyce, Cormac G. M. Gahan

**Affiliations:** 1 APC Microbiome Institute, University College Cork, Cork, Ireland; 2 School of Microbiology, University College Cork, Cork, Ireland; 3 School of Medicine, University College Cork, Cork, Ireland; 4 School of Biochemistry and Cell Biology, University College Cork, Cork, Ireland; 5 School of Pharmacy, University College Cork, Cork, Ireland; Nihon University School of Medicine, JAPAN

## Abstract

Disruptions to circadian rhythm in mice and humans have been associated with an increased risk of obesity and metabolic syndrome. The gut microbiota is known to be essential for the maintenance of circadian rhythm in the host suggesting a role for microbe-host interactions in the regulation of the peripheral circadian clock. Previous work suggested a role for gut bacterial bile salt hydrolase (BSH) activity in the regulation of host circadian gene expression. Here we demonstrate that unconjugated bile acids, known to be generated through the BSH activity of the gut microbiota, are potentially chronobiological regulators of host circadian gene expression. We utilised a synchronised Caco-2 epithelial colorectal cell model and demonstrated that unconjugated bile acids, but not the equivalent tauro-conjugated bile salts, enhance the expression levels of genes involved in circadian rhythm. In addition oral administration of mice with unconjugated bile acids significantly altered expression levels of circadian clock genes in the ileum and colon as well as the liver with significant changes to expression of hepatic regulators of circadian rhythm (including *Dbp*) and associated genes (*Per2*, *Per3* and *Cry2)*. The data demonstrate a potential mechanism for microbe-host crosstalk that significantly impacts upon host circadian gene expression.

## Introduction

All organisms have an autonomous biological clock with an oscillation cycle of approximately 24 hours that temporally regulates daily physiological events. In humans this circadian clock regulates sleep-wake cycles, body temperature and energy metabolism [1]. The master timekeeper resides in the neuronal cells of the suprachiasmatic nucleus (SCN) in the hypothalamus (known as the central clock) [1]. The central clock in turn influences circadian cycles in peripheral tissues through neuronal and hormonal signals (the peripheral clock). The duration of oscillation is not precisely 24 hours, so the central biological clock uses external environmental cues (mainly light and dark cycles) to reset and entrain circadian rhythms [2].

The basic cellular circadian clock comprises an autoregulatory transcriptional feedback loop with a regular cyclical oscillation [3]. The feedback loop is co-ordinated by the master regulator of circadian rhythm (Circadian Locomotor Output Cycles Kaput (CLOCK)), or a related protein NPAS2 which heterodimerize with ARNTL (BMAL1) and bind to the E box of the promoter of the *PER* and *CRY* genes to initiate their transcription. When PER and CRY accumulate in the cytoplasm they form a heterodimer, translocate into the nucleus and abolish CLOCK/ARNTL-mediated transactivation of their own gene transcription. The system is further regulated by RORα which activates *CLOCK* and *ARNTL* transcription, with another regulatory protein, REV-ERBα, acting to represses *ARNTL* expression. A further regulatory loop comprises the DBP protein which activates clock-controlled genes (through binding to D box elements), with E4BP4 competing for D box binding sites to repress gene transcription [1,3].

This autonomous cellular clock is expressed in all somatic cells and is entrained by the central clock and by other regulatory inputs [4]. It is well documented that food and feeding time [5] are important external cues which entrain the peripheral clock in most tissues. Any alteration in diet and feeding behaviour also affects microbial diversity in the host GI tract and studies have demonstrated that the gut microbiota is essential for maintenance of a regular peripheral circadian rhythm [6]. The mechanisms by which microbes may influence circadian rhythmicity in the host remain largely unclear. However recent work has demonstrated that short chain fatty acids are microbial signals that influence host circadian clock function and related metabolic parameters [7]. Understanding how microbes may influence circadian cycles is clinically significant as there is growing evidence that metabolic diseases such as obesity and glucose intolerance are closely linked to aberrations in circadian rhythmicity [8].

Bile acids represent important signalling molecules in the host as they interact with specific cellular receptors to regulate glucose, lipid and energy metabolism as well as immunological responses [9]. Bile acids are synthesised from cholesterol moieties in the liver and are subsequently conjugated to either taurine or glycine amino acids [10]. In the intestine these conjugated bile acids are deconjugated by bacteria expressing the bile salt hydrolase (BSH) enzyme to yield unconjugated bile acids which are then subject to further microbial modifications to generate secondary bile acids (deoxycholic acid (DCA) and lithocholic acid (LCA)) [11,12]. We recently demonstrated that elevated microbial BSH expression in the murine GI tract significantly increases local and systemic levels of unconjugated bile acids with profound effects upon lipid signalling pathways, weight gain and cholesterol levels in the host [13,14]. We also noted that genes involved in the regulation of circadian rhythm were influenced by elevated BSH in our study. Here we significantly extend these observations to show that microbe-generated unconjugated bile acids play a significant role in regulating control of the host circadian clock.

## Materials and Methods

### Cell culture

For cell culture work we utilised Caco-2 human epithelial colorectal adenocarcinoma cells [15] which are maintained by the APC Microbiome Institute cell culture collection and originally obtained from the American Type Culture Collection (Rockville). Caco-2 cells were grown in Dulbecco’s modified eagle medium (DMEM)(Sigma) supplemented with 10% foetal bovine serum (FBS)(Sigma) and 100 U/ml penicillin and 100 μg/ml streptomycin (Sigma) and 1% non-essential amino acids (Sigma). For *in vitro* circadian experiments, the Caco-2 cells were plated on 6 well plates and at ~80% confluency the cells were incubated in serum free DMEM-F12 (50:50) medium for 24 hours. At t = 0 the cells were serum shocked by exposing to DMEM-F12 (50:50) medium containing 50% FBS [4] and bile acid 100μM for 2 hours. The medium was then replaced with serum free DMEM and Ham F12 (50:50) medium with bile acids at a final concentration of 100μM. At the indicated times, the cells were washed once with PBS, lysed directly in lysis buffer (Fisher Scientific) and the lysates were stored at -70°C until used for RNA Isolation. All bile acids used in this study (Deoxycholic acid (DCA), Chenodeoxycholic acid (CDCA), Sodium taurodeoxycholate (TDCA) and Sodium taurochenodeoxycholate (TCDCA)) were obtained from Sigma. Cell viability was measured using the CellTiter-Glo Luminescent Cell Viability Assay (Promega) according to the manufacturer's instructions.

### Transepithelial resistance

Caco-2 cells were seeded on collagen-coated polycarbonate membrane cell culture inserts of diameter, 0.33 cm^2^ area with 0.4 μm pore size (Corning) at a density 250,000 cells/cm^2^ of cells in basal medium (BD biosciences). The following day the medium was changed to a differentiation medium (BD biosciences) and cells were allowed to differentiate for 2 days. Bile acid was added at the apical side when absolute TEER reached >350 ohms, which indicates an intact monolayer. The TEER was measured before and 24 hours after bile acid treatment using an EVOM—Epithelial Voltohmmeter (World precision instrument).

### Bile acid administration to mice

Eight week old male C57BL/6 mice used in this study were obtained from Harlan UK. Mice were acclimatized for 7 days in a controlled environment with a 12h: 12h light-dark cycle and divided into 3 groups. The first group of 8 mice was dosed by oral gavage with corn oil. A second and third group of 4 mice each were dosed by oral gavage with DCA (9 μmol/kg body weight) and CDCA (9 μmol/kg body weight) dissolved in corn oil respectively [16]. The mice were gavaged three times (t = 0, t = 24h and t = 48h) with corn oil or bile acids, were fasted for 3h to minimise inter-subject variation in bile acid profiles due to food intake during this time and euthanised to collect organs. All mice were euthanized within the same 45 minute period during the light phase. Liver, ileum and colon samples were stored in RNA later at -80°C.

### Ethics statement

This study was approved by the University Animal Experimental Ethics Committee (AEEC) and all animal experimental procedures used in this study were performed following institutional review and approval by the University Animal Experimental Ethics Committee (AEEC) (approval ID 2011/017) and under licence from the Irish Department of Health. All efforts were made to minimize suffering and to utilise to lowest possible number of animal subjects. None of the animals used in this study showed signs of pain or discomfort. At the relevant time-point mice were euthanized by cervical dislocation in accordance with institutional guidelines.

### cDNA synthesis

RNA was isolated from tissues using the RNeasy kit (Qiagen) according to the manufactures instructions and total RNA from cultured cells was isolated using a Nucleospin RNA kit (Fisher Scientific). cDNA synthesis was performed using 1 μg of total RNA isolated from tissues or from cells and mastermix containing a blend of random primer (Roche), RT enzyme (Roche) and dNTP (Roche)and real-time PCR amplification was performed as described previously [13] using a LightCycler^®^ 480 Real-Time PCR System (Roche, Dublin, Ireland). The results were normalised to the β actin gene and relative expression was calculated based on the 2^-ΔΔCt^ method as previously described [17]. All gene-specific primer sequences used in the study are listed in S1 and S2 Tables.

### Statistical analyses

Data analysis was carried out using MS excel and Graphpad prism 5 software (California, USA). Two technical replicates were carried out for each PCR reaction and for cell culture experiments values were calculated as standard error of the mean (SEM) from three independent biological experiments [18]. Multiple groups were analysed by one-way analysis of variance (ANOVA), followed by Dunnett’s multiple comparison post hoc test.

## Results

### Unconjugated bile acids influence the expression of circadian genes in cultured Caco-2 cells

Recent work has demonstrated that the microbiota is necessary to entrain the peripheral circadian clock [6]. Based upon previous findings from our group [13] we focused upon bile acids as potential signalling molecules in the regulation of expression of host circadian genes using a synchronised Caco-2 cell system that has been described previously [19]. As our previous work implicated the bacterial enzyme BSH in the expression of host clock-regulated genes [13] we tested the effects of unconjugated bile acids (DCA and CDCA) and corresponding conjugated bile acids (TDCA and TCDCA) upon expression of clock-regulated genes in the Caco-2 system. We exposed cells to concentrations of bile acids (100 μM) that have been proposed to be physiologically relevant and are non-toxic [20]. The CellTiter-Glo luminescent cell viability assay demonstrated that this concentration of bile acids has no cytotoxic effect on Caco-2 cells in our system (S1A Fig). The integrity of polarised Caco-2 monolayers as assessed by TEER measurements was also unaffected by bile acids at the concentrations used in this study (S1B Fig).

**Fig 1:**
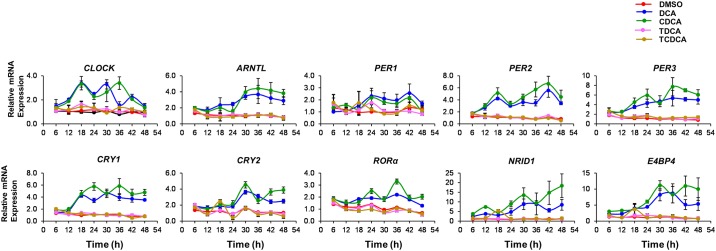
Microbially modified bile acids (DCA & CDCA) oscillate clock controlled genes in synchronised Caco-2 cells. Caco-2 cells were synchronized via serum starvation followed by a serum shock and treated with bile acids at 100 μM or with their corresponding bile salts. The cells were harvested for every 6h for a total of 48 hours. The relative expression levels of clock-regulated genes were measured using qRT-PCR and plotted in the graph versus time. The red colour graph represents the vehicle, pink represents the TDCA, brown represents TCDCA, blue represents DCA and green represents CDCA. Data represents pooled results from three independent biological replicates with two technical replicates.

We demonstrate that the unconjugated bile acids DCA and CDCA significantly increase the amplitude of the expression of cellular clock genes relative to conjugated moieties (TDCA and TCDCA) or control cells. The expression of *CLOCK* and *ARNTL* displayed a cyclical expression pattern that was amplified by the presence of unconjugated bile acids. *PER1* and *PER3* expression was also shown to cycle rhythmically but in a pattern antiphase to *CLOCK* and *ARNTL* in the presence of unconjugated bile acids, peaking at 24 h. Amplification of gene transcription was clearly associated with the presence of unconjugated bile acids and was not seen when cells were exposed to the corresponding conjugated bile acid moieties at equimolar concentrations (Fig 1).

**Fig 2:**
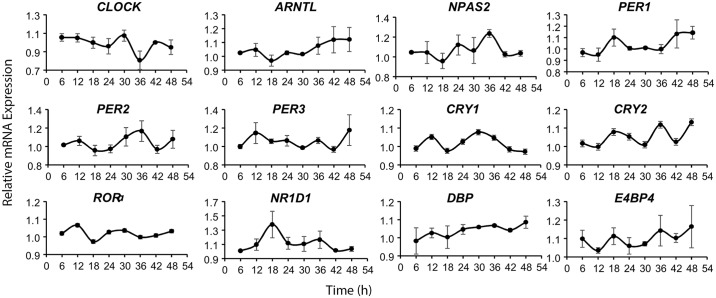
The periodic gene expression of CLOCK and CLOCK-controlled genes in synchronised Caco2 cells in the absence of bile acids. Caco2 cells were synchronized via serum starvation followed by a serum shock as outlined for Fig 1. The cells were harvested every 6h for a total of 48 hours. The relative expression levels of clock genes were measured using qRT-PCR and plotted in the graph versus time. Data represents pooled results from three independent biological replicates with two technical replicates.

Using a single sample time-point our previous study in germ free mice suggested that microbial BSH activity (which produces elevated concentrations of unconjugated bile acids) may affect the phase of oscillation of the circadian cycle [13]. Here we demonstrate that exposure of Caco-2 cells to unconjugated bile acids very subtly alters the phase of the circadian gene expression cycle. For instance expression of *PER2* in control synchronised cells peaks at 12, and 36 hours (see Fig 2 for baseline circadian oscillations in CACO-2 cells). In the presence of either DCA or CDCA this phase shifts towards a peak expression at 18 and 42 hours (Fig 1). Similar shifts in early peak expression occur for *PER1* and *PER3* from peak expression at 12/18 hours (Fig 2) to highest expression at 24 hours in the presence of unconjugated bile acids (Fig 1). The ability of unconjugated bile acids to affect the expression patterns of input regulators into the system (such as *RORα* or *E4BP4* (Fig 1) or *DBP* (S2 Fig)) may explain these alterations to the transcriptional cycle. Whilst the precise details surrounding the control of the cellular circadian gene expression cycle remain to be elucidated we clearly show that unconjugated bile acids influence the expression levels of genes involved in this cycle in cultured gut epithelial cells.

### Unconjugated bile acids influence expression of peripheral clock genes *in vivo*

Given that unconjugated bile acids influence the oscillation of circadian genes in cell culture we investigated whether oral administration of bile acids would influence expression of circadian genes in mice. We gavaged C57BL/6 mice with either DCA or CDCA (at 9 μmol/kg of body weight) dissolved in corn oil or with vehicle (corn oil) with three doses over a 48 hour period as per previous studies [16]. We did not administer mice with conjugated bile acids in this study as these would be subjected to *in vivo* deconjugation by the resident microbiota [21]. Mice were euthanized at the same single time point in order to allow direct comparison of patterns of gene expression across the different groups.

**Fig 3:**
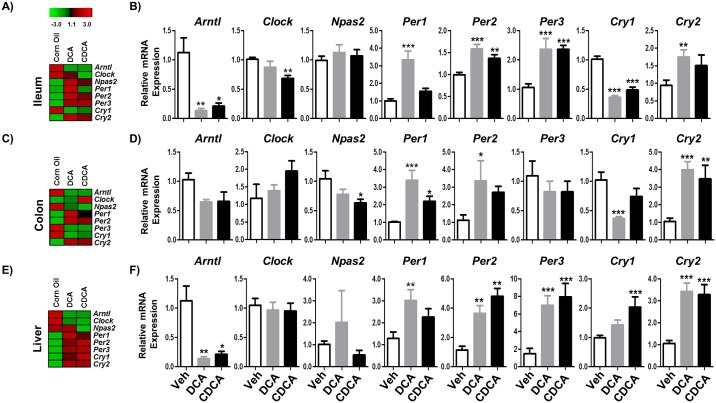
Unconjugated bile acids (DCA & CDCA) influence expression of clock related genes in murine peripheral organs. C57BL/6 mice were given vehicle (Corn oil) or DCA or CDCA each at 9 μmol/kg bodyweight dissolved in corn oil via oral gavage. The mice were gavaged three times (t = 0, t = 24h and t = 48h) with corn oil or bile acids and were fasted for 3h prior to harvesting of tissues from (A, B) Ileum, (C, D) Colon and (E, F) Liver. All tissues were harvested within the same 45 minute window during the light phase to minimize variation between subjects (see Materials & Methods). Total RNA was isolated from tissues and mRNA expression was measured using qRT-PCR. The expression of clock-regulated genes was analysed. Data are plotted relative to β-actin expression. The white bar represents the results from vehicle, grey bar represents the DCA treatment group and the black bar represents the CDCA group. Error bars denote SEM. Statistical significance determined by one way ANOVA (*P<0.05, **P<0.01, ***P<0.001), n = 4.

**Fig 4:**
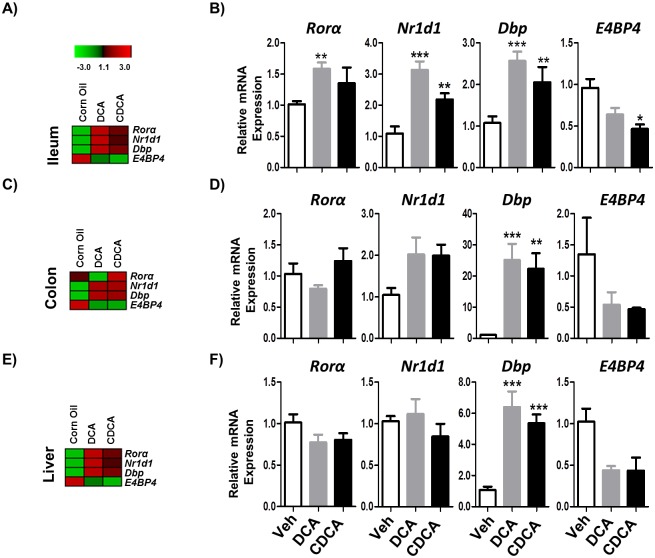
Unconjugated bile acids (DCA and CDCA) influence expression of genes encoding input regulators of clock-regulated genes in murine peripheral organs. C57BL/6 mice were given vehicle (Corn oil) or DCA or CDCA each at 9 μmol/ kg bodyweight dissolved in corn oil via oral gavage. The mice were gavaged three times (t = 0, t = 24h and t = 48h) with corn oil or bile acids and were fasted for 3h prior to harvesting of tissues from (A, B) Ileum, (C, D) Colon and (E, F) Liver. All tissues were harvested within the same 45 minute window during the light phase to minimize variation between subjects (see Materials & Methods). Total RNA was isolated from tissues and mRNA expression was measured using qRT-PCR. The expression of clock-controlled genes was analysed. Data are plotted relative to β-actin expression. The white bar represents the results from vehicle, grey bar represents the DCA treatment group and the black bar represents the CDCA group. Error bars denote SEM. Statistical significance was determined by one way ANOVA (*P<0.05, **P<0.01, ***P<0.001), n = 4.

Across all peripheral tissues (ileum, colon and liver) we noted that bile acid administration induces significant changes to circadian gene expression profiles which were broadly similar. In the ileum administration of DCA or CDCA bile acids significantly increased the expression of *Per1*, *Per2*, *Per3* and *Cry2* genes as compared to the vehicle (Fig 3A and 3B). As expected the expression of *Per* genes was antiphasic to *Clock* and *Arntl* which are on a different limb of the circadian gene expression cycle. Interestingly regulation of input regulators of the system was consistent with the observed changes in circadian gene expression (Fig 4A and 4B). We determined elevated expression of *Dbp* and reduced expression of *E4bp4*, changes that are predicted to increase expression of *Per* and related genes [1,3]. Both bile acids also stimulated expression of *Nr1d1* which encodes a factor that is known to block expression of *Clock/Arntl* [1,3].

Broadly similar patterns were seen in the colon where we determined significantly elevated levels of *Per1* and *Cry2* expression in mice administered DCA or CDCA with elevated levels of *Per2* expression in mice receiving DCA (Fig 3C and 3D). Again we determined that bile acid administration significantly elevated expression of the gene encoding the regulator *Dbp* with a trend towards reduction in *E4bp4* which may explain the enhanced expression of *Per* genes (Fig 4C and 4D).

As bile acids are efficiently absorbed in the terminal ileum we hypothesized that orally administered bile acids may influence the circadian expression of genes in the liver. Indeed we found that oral administration of DCA or CDCA to mice significantly altered the expression of hepatic circadian genes (Figs 3E and 3F and 4E and 4F). Bile acid administration increased expression of hepatic *Per2*, *Per3* and *Cry2* whilst DCA induced elevated *Per1* and CDCA induced elevated *Cry1* (Fig 3E and 3F). As expected these were in antiphase to the expression of *Arntl* which was significantly reduced in the liver. Again in this organ administration of DCA or CDCA significantly increased expression of *Dbp* a finding which potentially correlates with elevated *Per/Cry* expression profiles [1,3]. We appreciate that the work was carried out on a single timepoint in the murine circadian cycle. Further work in our laboratory is ongoing to determine the effects of bile acid and microbiota alterations across the wider circadian cycle in a murine model.

## Discussion

**Fig 5:**
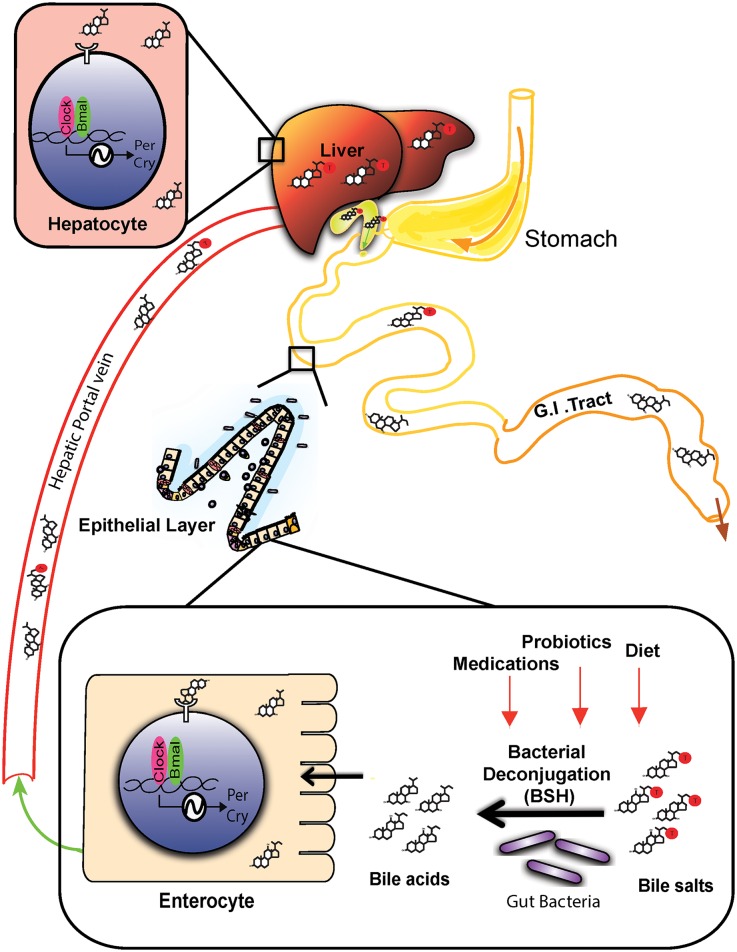
Model by which microbially-modified bile acids may influence expression of circadian genes. Bile acids are synthesised from cholesterol and conjugated with taurine or glycine in the liver and stored in the gall bladder. Upon food intake, the bile salts are released into the duodenum and aid in fat digestion and adsorption. Gut microbes in the intestinal lumen deconjugate bile salts to yield unconjugated bile acids. Recent work from other groups has demonstrated that diet, antibiotics and probiotics may influence this microbial activity (see discussion). Within the ileal enterocytes, unconjugated bile acids influence the amplitude and periodicity of circadian gene expression. Nearly 95% of bile salts and bile acids are reabsorbed in the terminal ileum and transported back to the liver via the hepatic portal circulation. Upon reaching the liver, the bile acids further influence circadian gene expression profiles.

Germ free [7,22] or antibiotic-treated microbiota-depleted mice [6] display altered oscillation of peripheral clock gene expression patterns with accompanying changes to diurnal metabolic profiles. This implies the existence of microbe-derived regulatory signals that influence circadian rhythm in host tissues. Recent studies have implicated microbe-associated molecular patterns (MAMP) and toll-like receptor interactions [6], production of short chain fatty acids, and H_2_S [7] as microbe-derived factors which influence host circadian rhythm. Here we demonstrate that bile acids generated through microbial activity in the gut (notably unconjugated primary and secondary bile acids) are potentially chronobiological signals that influence the circadian clock both locally in the gut, and in the liver (Fig 5). The work implicates gut bacterial enzymes including BSH (which metabolises conjugated bile acids to unconjugated bile acids) [21] and bile acid-inducible (Bai) enzymes (which metabolise primary bile acids to secondary bile acids) [12], as important regulators of the host circadian clock.

The current study extends previous work from our lab which demonstrated that the gut bacterial enzyme BSH significantly alters metabolic and circadian parameters in the host [13,14]. The study suggested that unconjugated bile acids generated through BSH activity represent signals that influence a range of physiological functions in the host. In the current study using a synchronised epithelial cell culture model we demonstrate that unconjugated bile acids DCA and CDCA are capable of significantly amplifying the expression of clock regulated genes. In contrast conjugated moieties (TDCA or TCDCA) at equimolar concentrations had no discernible effect on cellular circadian gene expression. Since unconjugated bile acids and secondary bile acids are generated only through microbial interactions in the gut [11,12] the data implicate microbial conversion of bile acids in the regulation of circadian gene expression patterns.

As bile acids are effectively recirculated via the enterohepatic portal system they have the potential to act as systemic signalling molecules in the host [9]. In our current study oral gavage of DCA or CDCA in mice influenced circadian gene expression profiles both locally in the gut (ileum and colon) and in the liver. The effect of bile acid administration was to increase expression of the gene encoding DBP, an activator of *Per/Cry* gene expression, with concomitant activation of *Per1*, *Per2*, *Per3* and *Cry2* expression, whilst expression of the gene encoding a competitive inhibitor E4BP4 was reduced. We accept that, as bile acids can alter the composition of the microbiota [23], the effects may be indirect. However the results suggest that intervention strategies using orally administered bile acids, bile acid sequestrants or BSH active microbial supplements may have the potential to alter systemic expression of clock-regulated genes in the host.

Several studies have shown that hepatic bile acid biosynthetic pathways are under circadian regulation [24,25]. Indeed knockdown of both *Per1* and *Per2* genes results in dysregulation of diurnal regulation of bile acid synthesis in mice [26]. Here we demonstrate that microbial metabolism of bile acids in the gut can potentially feed back to further influence peripheral circadian processes. Indeed whilst we acknowledge that further work is needed, we propose that any factor that alters microbial bile acid metabolism may have the potential to alter host circadian rhythm. For instance diet [27], antibiotics [28] or other medications [29] or probiotics [30] have been shown to alter microbial metabolism of bile acids through altered BSH activity and these perturbations may therefore have the potential to alter chronobiological signalling events through bile acid signalling. Previous studies have indicated a role for the farnesoid X receptor (FXR) in regulating the circadian rhythmicity of bile acid synthesis [31]. Further work will be necessary to determine the potential role of host bile acid receptors in the regulation of peripheral circadian rhythm and related processes.

Overall this study demonstrates a potential role for microbe-generated bile acids as chronobiological regulators of the peripheral circadian clock and indicates that intervention strategies which alter gut bile acids have the potential to influence the circadian clock. Understanding such feedback mechanisms is highly significant given the established links between desynchronization of circadian pathways and the development of obesity and metabolic disease in mice and humans [3,7,8].

## Supporting Information

S1 Fig Effect of bile acid on cell viability and membrane integrity. A) Caco2 cells were treated with bile acids DCA or CDCA or their corresponding bile salts at 100 μM or with vehicle for 24 hours. The percentage cell viability was determined and plotted in graph. B) Caco2 cells were seeded on the polycarbonate insert and allowed to form an intact monolayer and treated with 100 μM bile acid or their corresponding bile salts or vehicle for 24 hours. The transepithelial electrical resistance (TEER) was measure before and after bile acid treatment. For measuring membrane integrity, Caco-2 cells were seeded on collagen-coated polycarbonate membrane cell culture inserts of diameter, 0.33 cm^2^ area with 0.4 μm pore size (Corning) at a density 250,000 cells/cm^2^ of cells in basal medium (BD biosciences). The following day the medium was changed to a differentiation medium (BD biosciences) and cells were allowed to differentiate for 2 days. Bile acid was added at the apical side when absolute TEER reached >350 ohms, which indicates an intact monolayer. The TEER was measured before and 24 hours after bile acid treatment using an EVOM—Epithelial Voltohmmeter (World precision instruments).
(PDF)

S2 Fig Effect of bile acids DCA and CDCA on the DBP gene expression in synchronised Caco2 cells. Caco2 cells were synchronized via serum starvation followed by a serum shock and treated with bile acids at 100 μM or with their corresponding bile salts. The cells were harvested for every 6h for a total of 48 hours. The relative expression levels of *DBP* genes were measured using qRT-PCR and plotted in the graph versus time. The red colour graph represents the vehicle, pink represents the TDCA, brown colour represents TCDCA, blue represents DCA and green represents CDCA. Data represents from three independent biological replicates with two technical replicates.
(PDF)

S1 Table List of human primer and sequences used for qRT-PCR analysis. (PDF)

S2 Table List of mouse primer and sequences used for qRT-PCR analysis. (PDF)
