# Supplementary material for: Unconjugated Bile Acids Influence Expression of Circadian Genes: A Potential Mechanism for Microbe-Host Crosstalk
Source: PLoS One. 2016 Dec 1;11(12):e0167319. doi: 10.1371/journal.pone.0167319 (PMC5132238; doi:10.1371/journal.pone.0167319)
Supplement: S1 Fig — A) Caco2 cells were treated with bile acids DCA or CDCA or their corresponding bile salts at 100 μM or with vehicle for 24 hours. The percentage cell viability was determined and plotted in graph. B) Caco2 cells were seeded on the polycarbonate insert and allowed to form an intact monolayer and treated with 100 μM bile acid or their corresponding bile salts or vehicle for 24 hours. The transepithelial electrical resistance (TEER) was measure before and after bile acid treatment. For measuring membrane integrity, Caco-2 cells were seeded on collagen-coated polycarbonate membrane cell culture inserts of diameter, 0.33 cm2 area with 0.4 μm pore size (Corning) at a density 250,000 cells/cm2 of cells in basal medium (BD biosciences). The following day the medium was changed to a differentiation medium (BD biosciences) and cells were allowed to differentiate for 2 days. Bile acid was added at the apical side when absolute TEER reached >350 ohms, which indicates an intact monolayer. The TEER was measured before and 24 hours after bile acid treatment using an EVOM—Epithelial Voltohmmeter (World precision instruments). (PDF) [file pone.0167319.s001.pdf]

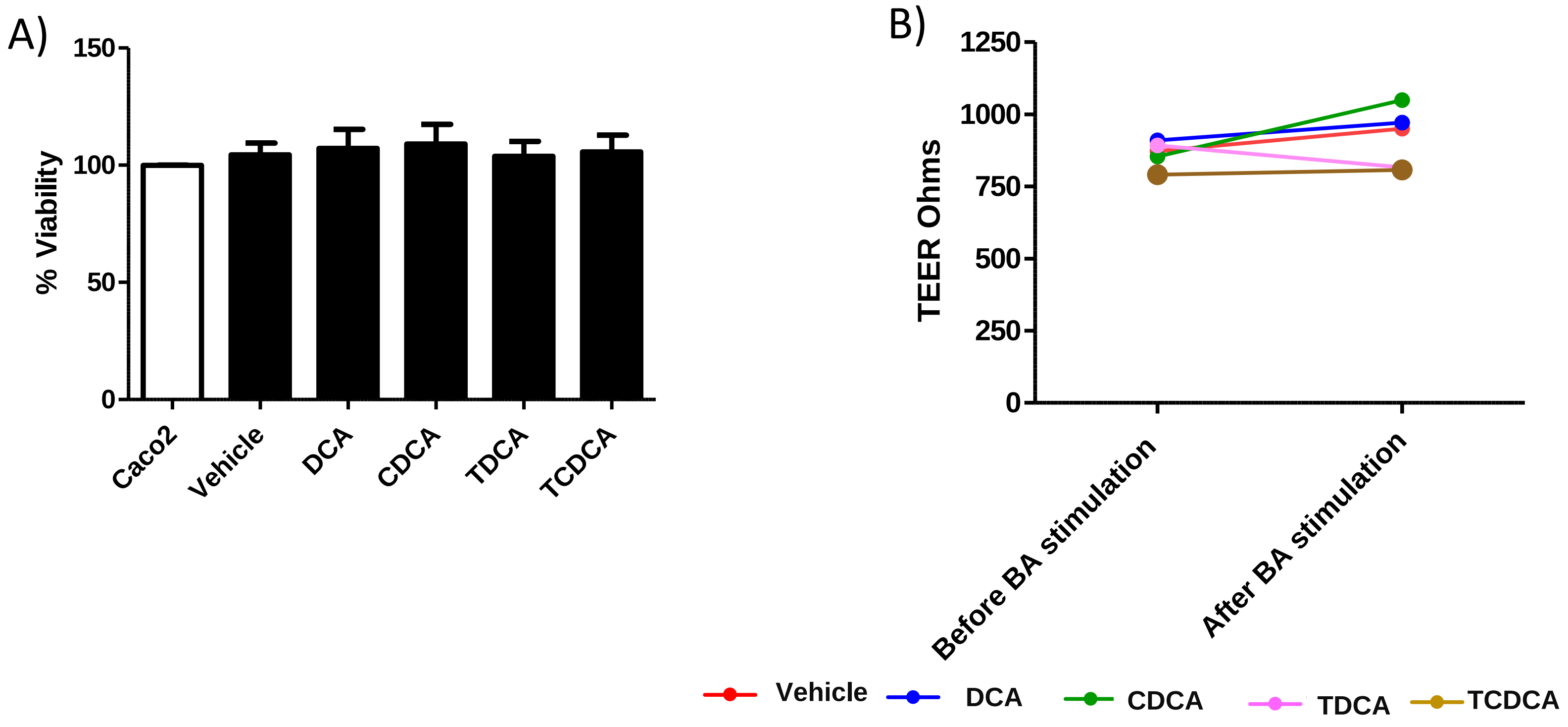

Supplementary Figure S1. Effect of bile acid on cell viability and membrane integrity. A) Caco2 cells were treated with bile acids DCA or CDCA or their corresponding bile salts at 100µM or with vehicle for 24 hours. The percentage cell viability was determined and plotted in graph. B) Caco2 cells were seeded on the polycarbonate insert and allowed to form an intact monolayer and treated with 100 µM bile acid or their corresponding bile salts or vehicle for 24 hours. The transepithelial electrical resistance (TEER) was measure before and after bile acid treatment. For measuring membrane integrity, Caco-2 cells were seeded on collagen-coated polycarbonate membrane cell culture inserts of diameter, 0.33 cm^2 area with 0.4 µm pore size (Corning) at a density 250,000 cells/cm^2 of cells in basal medium (BD biosciences). The following day the medium was changed to a differentiation medium (BD biosciences) and cells were allowed to differentiate for 2 days. Bile acid was added at the apical side when absolute TEER reached >350 ohms, which indicates an intact monolayer. The TEER was measured before and 24 hours after bile acid treatment using an EVOM - Epithelial Voltohmmeter (World precision instruments).
