# Supplementary material for: Unconjugated Bile Acids Influence Expression of Circadian Genes: A Potential Mechanism for Microbe-Host Crosstalk
Source: PLoS One. 2016 Dec 1;11(12):e0167319. doi: 10.1371/journal.pone.0167319 (PMC5132238; doi:10.1371/journal.pone.0167319)
Supplement: S2 Fig — Caco2 cells were synchronized via serum starvation followed by a serum shock and treated with bile acids at 100 μM or with their corresponding bile salts. The cells were harvested for every 6h for a total of 48 hours. The relative expression levels of DBP genes were measured using qRT-PCR and plotted in the graph versus time. The red colour graph represents the vehicle, pink represents the TDCA, brown colour represents TCDCA, blue represents DCA and green represents CDCA. Data represents from three independent biological replicates with two technical replicates. (PDF) [file pone.0167319.s002.pdf]

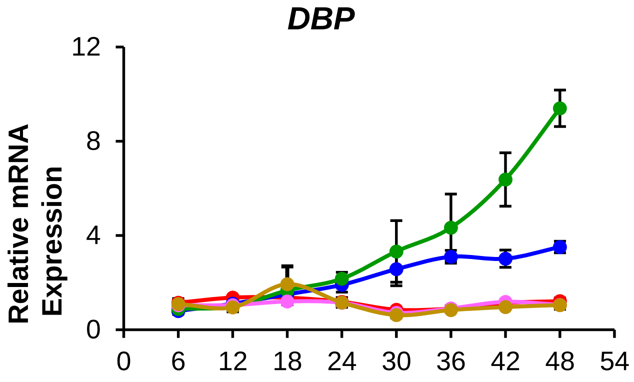

Supplementary Figure S2. Effect of bile acids DCA and CDCA on the DBP gene expression in synchronised Caco2 cells. Caco2 cells were synchronized via serum starvation followed by a serum shock and treated with bile acids at 100 µM or with their corresponding bile salts. The cells were harvested for every 6h for a total of 48 hours. The relative expression levels of *DBP* genes were measured using qRT-PCR and plotted in the graph versus time. The red colour graph represents the vehicle, pink represents the TDCA, brown colour represents TCDCA, blue represents DCA and green represents CDCA. Data represents from three independent biological replicates with two technical replicates.
