# Supplementary material for: Unconjugated Bile Acids Influence Expression of Circadian Genes: A Potential Mechanism for Microbe-Host Crosstalk
Source: PLoS One. 2016 Dec 1;11(12):e0167319. doi: 10.1371/journal.pone.0167319 (PMC5132238; doi:10.1371/journal.pone.0167319)
Supplement: S1 Table — (PDF) [file pone.0167319.s003.pdf]

Supplementary Table S1. List of human primer and sequences used for qRT-PCR analysis

Human primer	Sequence (5'→3')
hClock_L	gagagcgcgaaggaaatct
hClock_R	gcagctttgcaggaacaagta
hArntl_L	caggaaaaataggccgaatg
hArntl_R	gcgatgaccctcttatcctg
hPer1_L	ggacactcctgcgaccag
hPer1_R	gggagtgaggtggaagatctaa
hPer2_L	gactgcaaacctggcacttc
hPer2_R	gtgtctgagggttcatcacg
hPer3_L	gcgcattctcatgacatacc
hPer3_R	tgctgctgcctcatactttc
hCry1_L	catcctggaccccctggtt
hCry1_R	caagacactgaagcaaaaatcg
hCry2_L	gagcaaggatgcctgagact
hCry2_R	cggtgatcagcttctctgc
hRORα_L	gcattattttctgcatttgtactga
hRORα_R	tgcagtttttcaatttttaccttttc
hNR1D1_L	aactccctggcgcttacc
hNR1D1_R	gaagcggaattctccatgc
hE4BP4_L	cgcccctttctttctcct
hE4BP4_R	agttgggcctccttcgttat
hACTB _L	attggcaatgagcggttc
hACTB _R	cgtggatgccacaggact
hNpas2_L	caggctatgactactaccacattga
hNpas2_R	ttgccaaactgcatcagg
hDbp_L	aggcaagaaaaatccaggtg
hDbp_R	gcctcgttgttcttgtaccg
