# Supplementary material for: Unconjugated Bile Acids Influence Expression of Circadian Genes: A Potential Mechanism for Microbe-Host Crosstalk
Source: PLoS One. 2016 Dec 1;11(12):e0167319. doi: 10.1371/journal.pone.0167319 (PMC5132238; doi:10.1371/journal.pone.0167319)
Supplement: S2 Table — (PDF) [file pone.0167319.s004.pdf]

Supplementary Table S2. List of mouse primer and sequences used for qRT-PCR analysis

Mouse primer	Sequence (5'→3')
mClock_L	ccagtcagttggtccatcatt
mClock_R	tggctcctaactgagctgaaa
mNpas_L	ggtccaggactccaacgtc
mNpas_R	tgctgaagctcagggacac
mArntl_L	agtacgcctccccctgat
mArntl_	tgtctggagtccctccattt
mPer1_L	gcttcgtggacttgacacct
mPer1_R	tgctttagatcggcagtggt
mPer 2_L	atcgtgaagaacgcggata
mPer 2_R	caggatcttcccagaaacca
mPer 3_L	cggagagtatgtcattctggatt
mPer_3_R	tggtggcaaaaacatcttca
mCry1_L	atcgtgcgcatttcacatac
mCry1_R	tccgccattgagttctatgat
mCry2_L	gcagagcctggttcaagc
mCry2_R	gccactggatagtgctctgg
mRorα_L	cctactgttccttcaccaacg
mRorα_R	atgttctgggcaaggtgttc
mNr1d1_L	ccccaggaagtctacaagtgg
mNr1d1_R	agcaccatgccattcagc
mDbp_L	gcctctgagcgacaggac
mDbp_R	aaggctcctcagcccaag
mE4BP4_L	agggagcagaaccacgataa
mE4BP4_R	ccggatggaggagacaaat
